# Genomic Variation Prediction: A Summary From Different Views

**DOI:** 10.3389/fcell.2021.795883

**Published:** 2021-11-25

**Authors:** Xiuchun Lin

**Affiliations:** College of Information and Electrical Engineering, China Agricultural University, Beijing, China

**Keywords:** genome, variation, machine learning, genomic mutation, prediction

## Abstract

Structural variations in the genome are closely related to human health and the occurrence and development of various diseases. To understand the mechanisms of diseases, find pathogenic targets, and carry out personalized precision medicine, it is critical to detect such variations. The rapid development of high-throughput sequencing technologies has accelerated the accumulation of large amounts of genomic mutation data, including synonymous mutations. Identifying pathogenic synonymous mutations that play important roles in the occurrence and development of diseases from all the available mutation data is of great importance. In this paper, machine learning theories and methods are reviewed, efficient and accurate pathogenic synonymous mutation prediction methods are developed, and a standardized three-level variant analysis framework is constructed. In addition, multiple variation tolerance prediction models are studied and integrated, and new ideas for structural variation detection based on deep information mining are explored.

## Introduction

The decreasing cost of genome sequencing has resulted in a large amount of sequence information and variation data becoming available. According to the number of mutated bases, genomic variations have been classified as: 1) single-nucleotide variations (formerly single-nucleotide polymorphisms); 2) very short insertions and deletions, usually less than 50 bp; and 3) structural variations, usually longer than 50 bp (Genome structural variati, 2011). Gene mutations are known to be closely related to the occurrence and development of diseases (Hunt et al., 2014; Alkan et al., 2011; Yin et al., 2020; Fang et al., 2019; Li et al., 2020a; He et al., 2020; Zhang et al., 2020a; Zhang et al., 2016; Hu et al., 2021; Hu et al., 1990; Hu et al., 2020). High-throughput sequencing technologies have allowed the mutations in the genomes of patients with particular diseases to be determined systematically, quickly and accurately, including common but less studied synonymous mutations in the coding regions of genomes (Meyerson et al., 2010; Li et al., 2017; Cheng et al., 2018; Zhou et al., 2019). Synonymous mutations are single-nucleotide mutations that occur in the coding regions of genes but do not change the amino acid sequence of the protein due to the degeneracy of the genetic code. Because they do not change the coded amino acids, synonymous mutations were once mistakenly thought to have no biological function (Hong et al., 2020; Tang et al., 2020; Cheng et al., 2021a). However, later systematic studies have shown that synonymous mutations are involved in a variety of biological processes and play important roles in the occurrence and development of diseases (Li et al., 2020b; Yang et al., 2021a). Whole genome sequencing using reversible terminator chemistry can generate accurate nucleotide sequences of billions of bases at low cost (Bentley et al., 2008), which greatly improves the data obtained in sequencing projects. Structural variations in genomes are closely related to the occurrence and development of many diseases that affect human health. Therefore, the detection of structural mutations is essential to understand the mechanisms of diseases, find pathogenic targets, and carry out personalized precision medicine (Guo et al., 2011; Liu et al., 2021a; Yu et al., 2021a; Yu et al., 2021b). However, detecting structural variations can be difficult, and therefore methods that can accurately and quickly predict genomic variations are urgently required.

**FIGURE 1:**
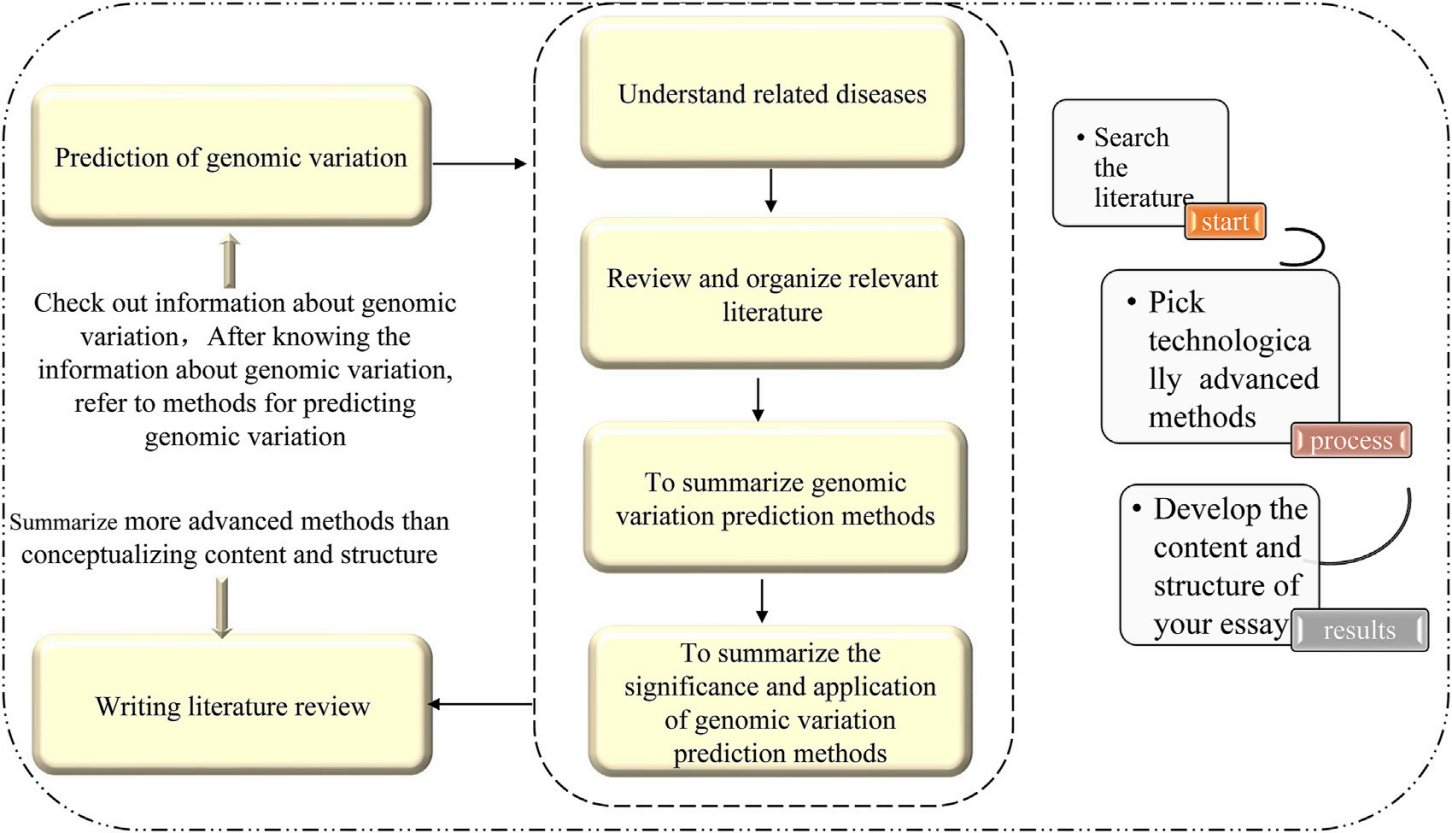
Idea map of the paper.

In this paper, an efficient and accurate method for predicting disease-causing synonymous mutations based on existing research and using machine learning theories and methods is reviewed. This method not only incorporates the current understanding of the pathogenic mechanism of synonymous mutations, but also provides a theoretical basis for the diagnosis and treatment of diseases, drug development, and the development of memory precision medicine. There are three steps to this method: 1) defining a standardized variation analysis framework based entirely on genome sequencing data; 2) using computational methods to construct a variation tolerance prediction model as the classification basis, and two high-performance variation mechanisms (influence of protein solubility and metabolic stability) prediction models; and 3) developing software tools and combining multiple models to provide general variation analysis services, as well as medical research and precision medical services. For structural variation detection based on deep mining of information, the following key technologies and methods are established: 1) extract sequence features related to structural variations from different sides and establish a comprehensive representation of variation; 2) use the sequence to comparison text information and the corresponding variation feature map to generate images and amplify imbalanced images of small samples; and 3) using the powerful feature representation capabilities of deep learning, automatically extract global features, hidden features, and associated features to complete variation detection. This approach will be an effective way to improve the accuracy of genome structural variation detection, and will also help to promote the development of new structural variation detection technologies. The outline of this study is shown in Figure 1.

## Genomic Variation Prediction Methods

Variations in the human genome are related to human evolution and disease risk (Jiang and Liu, 2016; Jiang et al., 2017; Liu et al., 2017; Liu et al., 2018a; Liu et al., 2018b; Liu et al., 2018c; Liu et al., 2019; Wu et al., 2019; Xu et al., 2019; Deng et al., 2021). Moreover, with the systematic in-depth studies of single-nucleotide mutations, especially those that have special genetic variation patterns such as synonymous mutations, the understanding of the composition of the human genome, genetic differences between individuals, and the pathogenic mechanisms of diseases has greatly improved. The genetic variation prediction method reviewed in this paper will help to make such studies more convenient and economical by identifying variations of interest that can be targeted (Table 1).

**TABLE 1:** Summary of genomic variation prediction methods.

Type	Methods	Algorithm
Pathogenic synonymous mutations	SilVA (Buske et al., 2013)	Random forest
	DDIG-SN(Livingstone et al., 2017)	Support vector machine
	regSNPs-splicing (Zhang et al., 2017a)	Random forest
	Syntool (Zhang et al., 2017b)	—
	TraP (Gelfman et al., 2017)	Random forest
Genome sequencing	CADD (Kircher et al., 2014)	Support vector machine
	MutationTaster2 (Cooper, 2014)	Naive Bayes
	Mut-Pred (Li et al., 2009)	Random forest
	PolyPhen-2 (Adzhubei et al., 2010)	Naive Bayes
	PON-P2 (Niroula et al., 2015)	Random forest
	VEST (Carter et al., 2013)	Random forest
Deep mining of structural variation information	DeepBind(Alipanahi et al., 2015)	deep learning
	DeepVariant (Angermueller et al., 2017)	deep neural networks
	DeepCpG (Poplin et al., 2018)	deep neural networks

### Pathogenic Synonymous Mutations

In recent years, the interest and attention of researchers in the analysis and prediction of pathogenic synonymous mutations have increased. Published methods include SilVA (Buske et al., 2013), DDIG-SN (Livingstone et al., 2017), regSNPs-splicing (Zhang et al., 2017a), Syntool (Zhang et al., 2017b) and TraP (Gelfman et al., 2017). sHowever, the available prediction methods still have certain defects that need improvement. Among them, the use of machine learning methods to predict pathogenic synonymous mutations is still in the preliminary stage. The main problems that remain to be solved include: 1) positive sample data is scarce and standard negative sample data is lacking (Zhang et al., 2020b); 2) feature representation ability is weak and not easy to promote (Buske et al., 2013; Wei et al., 2018; Xiong et al., 2018; Jin et al., 2019; Shen et al., 2019; Su et al., 2019; Wei et al., 2019; Yang et al., 2020a; Zhang et al., 2020c; Peng et al., 2020; Su et al., 2020; Teng et al., 2020; Chu et al., 2021a; Cheng et al., 2021b; Chu et al., 2021b; Jin et al., 2021; Su et al., 2021); and 3) the prediction performances of existing methods need to be improved, and the results of different methods have a low degree of coincidence (Cheng et al., 2019). The methods reviewed in this article aim to solve these problems.

The aim of this project is to develop an efficient and accurate method for predicting disease-causing synonymous mutations. The main steps are as follows: 1) establish a data set using a variety of different methods and data sources; 2) analyze in detail the biological characteristic attributes related to pathogenic synonymous mutations; 3) design machine learning methods to predict mutations; and 4) develop a public service platform and corresponding software system to predict disease-related mutations. The following aspects were included in the method. 1) A pathogenic synonymous mutation database and benchmark data set are constructed. Then, pathogenic synonymous mutation data reported in the literature are collected to supplement the database, and the two types of data are integrated to improve the database. 2) A feature representation method of the pathogenic mechanism of the synonymous mutation is established. Synonymous mutations can occur in various processes of gene expression. In this paper, the pathogenic principle of synonymous mutations was fully utilized, and the method of numerical expression of pathogenic synonymous mutations was studied at the DNA, RNA, and protein levels. In addition, the same data set and the same machine learning model are combined for testing, and then the feature selection method is used to remove irrelevant features from the extracted features to select a relatively good feature representation method. 3) A prediction method for pathogenic synonymous mutations with convolutional neural network was used as the basic model. The aim was to learn the representation method of pathogenic synonymous mutation data based on deep learning, especially the efficient implicit feature representation ability. The deep feature representation method of the biological characteristics of pathogenic synonymous mutations is also used to make up for the lack of feature representation ability of shallow learning. Then, deep network structure design and deep model training optimization strategies are studied to improve the robustness and generalization performance of the model. 4) The prediction method of pathogenic synonymous mutation based on ensemble learning is evaluated. To reduce the correlation of individual classifier results, a method suitable for training and learning is selected from the existing prediction methods to obtain the individual classifier. Then, randomization is introduced in the learning and training processes to obtain diverse individual classifiers. Finally, a learning algorithm for the integrated decision-making classifier is designed to construct a suitable secondary classifier to more effectively solve the problem of pathogenic synonymous mutation prediction. How to perform ensemble pruning after the generation of cascading ensemble classifiers should also be studied to further improve the predictive performance of the cascading ensemble learning classifier and obtain better pathogenic synonymous mutation predictions. 5) Result verification and algorithm software development are performed. The predictive analysis data obtained in 3 and 4 above are analyzed and the results with high reliability and potential clinical application value are selected to carry out molecular and biochemical experiments to determine the biological functions of synonymous mutations at the cellular level and to verify the accuracy of the prediction results.

### Genome Sequencing

Methods to rapidly and comprehensively interpret the various new variations identified in genome sequencing are lacking. Therefore, it is not yet possible to associate variations with possible diseases or health issues, which greatly reduces the value of genome sequencing. A primary task of sequencing research is how to analyze the sequence data, especially the variation information that it contains. Prediction methods can effectively solve this problem (Castrense et al., 2019; Gang et al., 2019; Liu et al., 2020a; Jin et al., 2021; Yin et al., 2021). The published methods include CADD (Kircher et al., 2014), MutationTaster2 (Cooper, 2014),Mut-Pred (Li et al., 2009),PolyPhen-2 (Adzhubei et al., 2010),PON-P2 (Niroula et al., 2015) and VEST (Carter et al., 2013). The aim of the project was to design a standard variation analysis framework for genomic variation prediction. Specifically, a variation tolerance prediction model is constructed based on the genome sequencing data and calculation method, and a relatively high-performance variation mechanism is also constructed based on the influence of protein solubility and metabolic stability. The following aspects are included. 1) A general prediction model of variation tolerance classification is constructed based on the existing variation data for all kinds of variations (e.g., replacement, insertion, and deletion) using only the sequence information as the basic and important step of the analysis services. A highly accurate predictive model ProtSol is constructed. New data will be collected and sorted out, the training data set will be integrated, and input features will be selected. Then, the classification algorithm will be optimized and a protein solubility impact prediction model with higher accuracy and greater generalization will be established for variation analysis. 3) A model ProtMS to predict the effect of variation on protein metabolism stability is constructed. Classification and regression models based on sequence information will be established to predict the impact of variations on metabolic stability. These models will serve as important parts of the mechanism analysis. In addition, browser/server architecture variant analysis software tools will be developed and released to provide online services for researchers and clinical medical staff.

### Deep Mining of Structural Variation Information

**FIGURE 2:**
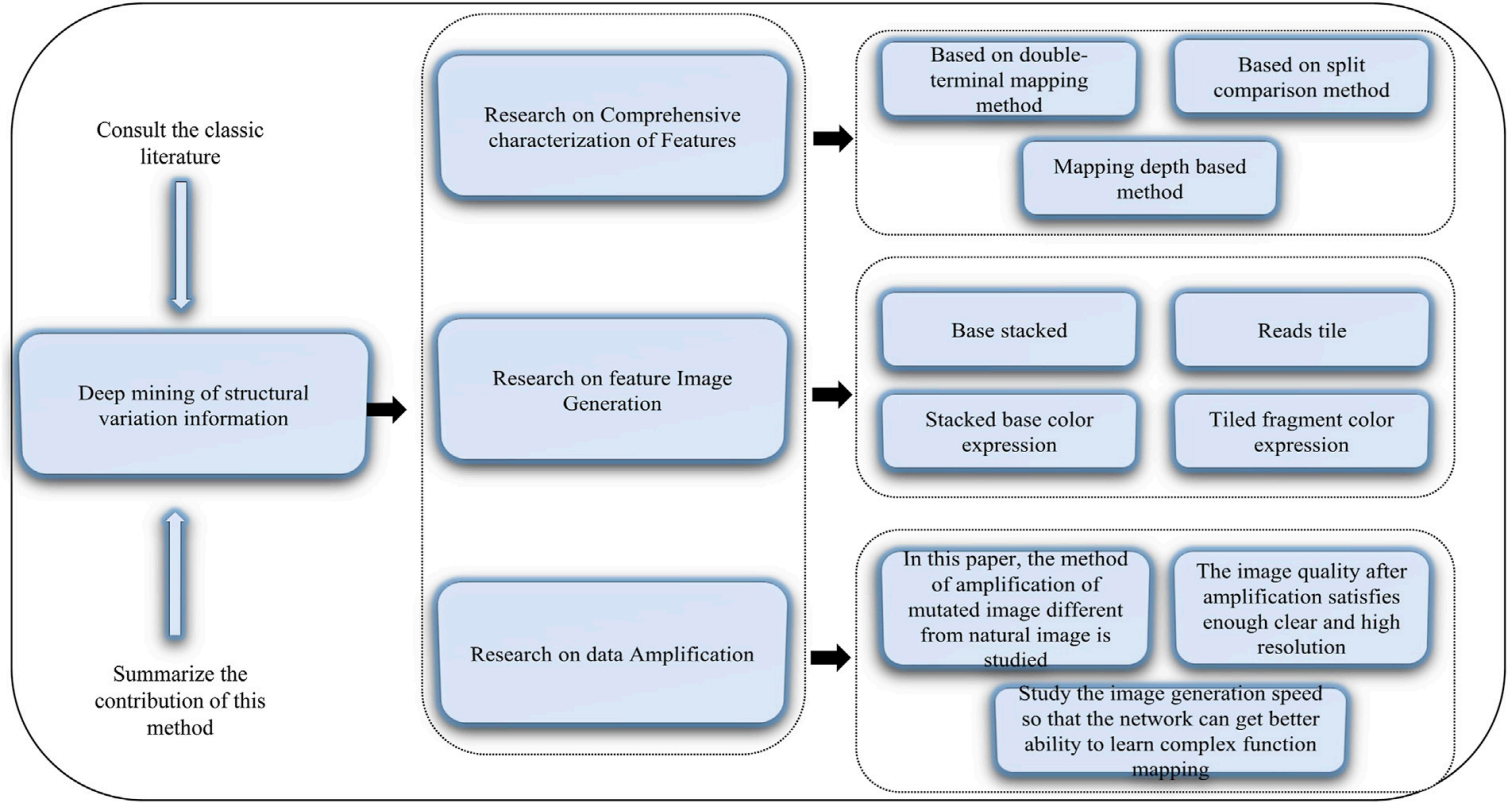
Diagram of deep mining of structural variation information.

Structural variations in the human genome can cause diseases (Feuk, et al., 2006). Structural variations include translocation, inversion, deletion, and duplication of genes, and accurate detection of genetic variations or genetic testing can contribute to the exploration and analysis of diseases and life processes. The obtained structural variant information can be applied, for example, to target drugs to tumors and to provide a reliable reference for clinical applications (Xu et al., 2018; Xue et al., 2018; Tang et al., 2019; Liu et al., 2020b; Zhang et al., 2020d; An and Yu, 2021; Liu et al., 2021b; Wang et al., 2021; Wu and Yu, 2021). Therefore, we need to mine structural variation information accurately. Published methods include DeepBind (Alipanahi et al., 2015), DeepVariant (Angermueller et al., 2017), and DeepCpG (Poplin et al., 2018). Deep mining of the human genomic structural variation information includes the following aspects. 1) Comprehensive characterization of features using three basic detection methods: sequence features related to structural variation from different aspects, definition of representation modes of different types of variation, and construction of classification and combined expression of feature descriptions. Then, the comprehensive characteristics are obtained by combining three types of detection methods (i.e., double-terminal mapping-based, split-comparison-based, and mapping depth-based methods) to form a complete comprehensive characterization of variation features. 2) Feature image generation whereby images are generated to discover comprehensive variation information from two main aspects, pixel composition and color expression. Pixel composition includes base stacking and fragment tiled, and color expression includes stacked base color expression and tiled fragment color expression. 3) Data amplification to ensure that the generated images can be used for deep learning training and recognition. The main purpose of data amplification is to avoid the problem of network overfitting or a decline in the recognition of minority samples. This step includes mainly studying the method of amplification of mutated images different from natural images; studying sufficiently clear and high-resolution amplified images, and studying the speed of image generation to ensure the network is better able to learn the mapping of complex functions. A schematic diagram of this method of deep mining of structural information is given in Figure 2.

## Literature Contribution

Early studies of the human genome focused mainly on collecting data and understanding structural variation at the genome level. In this paper, the literature closely related to diseases and current medical fields was reviewed, including genome prediction methods and many aspects of genome variation. The genomic variants associated with the diseases are summarized in Table 2. Bioinformatics analysis studies of pathogenic synonymous mutations aim to integrate different data sources and numerical types and detect reliable characteristics for feature representation methods, and also to accurately characterize feature coding methods that are intrinsically linked to pathogenic synonymous mutations. For ensemble learning methods, it is necessary to propose a pruning mechanism for the primary classifier learning algorithm with adaptive learning ability. Genome sequencing studies aim to establish a set of standardized solutions and integrate existing databases and multiple prediction models. The goal is to solve the versatility and generalization of variation analysis models, to solve the data imbalance problem that is common in variation data, and to find sequence-based biological characteristics in different variation prediction models. The contribution of the deep mining of structural variation information is to develop a new way of generating variation feature images from sequence comparison text information, a method of data amplification for small samples of variation images to achieve a balance, and an accurate detection technology framework by digging deep into the genomic structural variation information. The proposed points can take genomic variation prediction research one step further, and provide medication recommendations for the treatment of specific diseases, thereby reducing the adverse effects on patients due to improper medication strategies. This is one of the reasons why genomic variation is an important area of study.

**TABLE 2:** Summary of genomic variations associated with disease.

Disease	Causes	Result
Type 2 diabetes (Freemantle et al., 2005)	There were 139 common gene variants and 4 rare gene variants	Availability of Inhaled Insulin Promotes greater perceived acceptance of insulin therapy in Patients with type 2 diabetes
Neonatal epilepsy (Thuresson et al., 2016)	Whole gene repeats of SCN2A and SCN3A	Extra copy of SCN2A has an effect on epilepsy pathogenesis
Bladder cancer (Bonberg et al., 2013)	Copy number variation in GSTM1 gene	A loss of 9p21 was less predictive for detecting bladder cancer
Lung cancer (Yang et al., 2013)	Cnv-67048 variation on WWOX	be related with altered WWOX gene expression and exons absence in them
A wide variety of tumor (Abdel-Rahman et al., 2011)	BAP1 mutation	BAP1 is the candidate gene in only a small subset of hereditary UM, suggesting the contribution of other candidate genes.

## Conclusion

The study of structural variations in genomes can promote research on genome evolution, significant biological phenotypic changes (Yang et al., 2020b; Yang et al., 2020c; Yin et al., 2020), the treatment of many diseases (Li et al., 2018; Yang et al., 2021b; Long et al., 2021), and recommendations for therapeutic drugs (Wei et al., 2014; Ding et al., 2020a; Ding et al., 2020b; Wang et al., 2020; Wei et al., 2020). The accurate prediction of genomic variation is of great importance to studies of many diseases, which indicates the significance of this literature review through which existing variation data were integrated and collected, and a tolerance classification model of various variations was constructed based on sequence information. Furthermore, all the literature is experimenting around key scientific issues. It is essential to accurately predict individual genomic variation events that are conducive to systematically inferring the process of variation formation, so that the results can be confidently used for the clinical application of precision medicine. Finally, accurate predictions also help in analyzing the functions of synonymous mutations and can guide relevant experiments. Therefore, genomic variation prediction is of great significance to drug design and precision medicine.
